# The Bias Towards Disease

**Published:** 1920-08-14

**Authors:** 


### THE MATRONS' AND SISTERS' DEPARTMENT.

### THE BIAS TOWARDS DISEASE.

A veritable gauntlet has been thrown down into the arena by Miss Cooper-Hodgson, Superintendent Health Visitor in Durham. Her daring assertion that hospital training as at present carried on is not the best preparation which a woman can have for the career of health-visiting fell like a challenge into the serene air of the Royal Sanitary Institute Congress. It is no use to meet it with a flat denial. The issues are too serious for this easy controversial method to avail. Nor can the point be settled by bringing forward on either hand conspicuous successes or failures of hospital nurses as health visitors. The root of the controversy lies much deeper. It is a call for searching examination into the aims of hospital training and the probable effect on young minds of the three or four years spent in the wards.

We must plainly say that Miss Cooper-Hodgson has brought forward nothing so far to justify her contention that hospital training harms the woman who aspires to the career of health visitor. But she has said both too much and too little. It is fair to the profession against which she has made definite charges that nursing superintendents should be told what vital defect is observable in the women they turn out of the training schools which puts them in a worse position as regards learning the duties of health-visiting than if they had come straight from their own homes or from university life.

The health visitor is mainly though not exclusively occupied in keeping children under observation from a period shortly after birth and assisting their mothers by advice, etc., so far as lies in her power, to rear their offspring in the right way. Should the visitor be possessed of a bias towards disease it would presumably show itself either in neglect of the healthy babies or in a morbid inclination to see disease where it is absent. We may dismiss the former danger as negligible. The health visitor naturally finds little to do in the homes of the healthy where the mother is proving her competence to rear her own children. The bias towards disease must display itself, then, in a tendency towards unnecessary fuss, towards frightening the mother into the belief that all is not going well and thus bringing an atmosphere of suspicion and apprehension round the child. Is this the habit of mind engendered by three years spent in the healing of the sick?

We may as well look facts squarely in the face and realise that the habit of mind which regards illness as a kind of malign fate ready to swoop down at any minute without ascertainable cause is common to nearly every one in the present day. Both the lay health visitor and the probationer before beginning her course are liable to be tainted with it, and it flourishes among mothers of every degree. It is ridiculous to accuse hospital training of imparting it, but is there anything in hospital training which eradicates it? If our trained nurse pass out of the wards without learning to believe in the forces which make for health, she must be regarded from that point of view as a failure, the product of a bad system.

There is much, and very much, in the modern system of medicine and surgery to lend the nurse that supreme confidence in the innate forces of health which radiates well-being wherever it makes itself felt. She continually observes bad symptoms disappear, often under little else than the restoration of good conditions, the regulation of diet and such natural remedies as the application of warmth or rubbing. She watches the healing of wounds "by first intention" or after the surgeon's skill has removed the impediments which obstruct the healing process. And the nearer she comes to the mind of the physicians and surgeons under whom she works, the closer she finds herself to the healing forces which lie outside the pharmacopœia, but form the great foundation on which the highest skill depends for cure. If anywhere the lesson of dependence on modern hygiene is to be learned, it should be in the wards wherein those who have broken down from ignorance or the pressure of circumstances learn to use aright the natural powers they possess and regain their place among the healthy. Yet it is by no means certain that nurses have these truths put before them in a form which they can assimilate. We look in vain in the curricula of lectures delivered during term time for the assertion and development of principles of health. If the nurse does not believe in God's gift of health to His people she is of all women most miserable. Is our armour strong enough to resist the arrow which Miss Cooper-Hodgson, reinforced by Professor Kenwood, has levelled at the profession? Much more than the future of health-visiting is at stake.

